# Cholangiocarcinoma with respect to IgG4 Reaction

**DOI:** 10.1155/2014/803876

**Published:** 2014-07-15

**Authors:** Kenichi Harada, Yasuni Nakanuma

**Affiliations:** ^1^Department of Human Pathology, Kanazawa University School of Medicine, Kanazawa 920-8640, Japan; ^2^Department of Pathology, Shizuoka Cancer Center, Shizuoka 411-8777, Japan

## Abstract

IgG4 reactions marked by infiltration of IgG4-positive plasma cells in affected organs occur in cancer patients and in patients with IgG4-related diseases. Extrahepatic cholangiocarcinomas including gall bladder cancer are often accompanied by significant IgG4 reactions; these reactions show a negative correlation with CD8-positive cytotoxic T cells, suggesting that the evasion of immune surveillance is associated with cytotoxic T cells. The regulatory cytokine IL-10 may induce IgG4-positive plasma cell differentiation or promote B cell switching to IgG4 in the presence of IL-4. Cholangiocarcinoma cells may function as nonprofessional antigen presenting cells that indirectly induce IgG4 reactions via the IL-10-producing cells and/or these may act as Foxp3-positive and IL-10-producing cells that directly induce IgG4 reactions. Moreover, IgG4-related disease is a high-risk factor for cancer development; IgG4-related sclerosing cholangitis (IgG4-SC) cases associated with cholangiocarcinoma or its precursor lesion biliary intraepithelial neoplasia (BilIN) have been reported. IgG4-positive cell infiltration is an important finding of IgG4-SC but is not a histological hallmark of IgG4-SC. For the diagnosis of IgG4-SC, its differentiation from cholangiocarcinoma remains important.

## 1. Introduction

Inflammatory biliary diseases with periductal fibrosis are categorized as sclerosing cholangitis. In addition to the prototype of sclerosing cholangitis, primary sclerosing cholangitis (PSC), IgG4-related sclerosing cholangitis (IgG4-SC) is categorized as sclerosing cholangitis. Although IgG4-SC is characterized by the infiltration of numerous IgG4-positive cells in the wall of bile ducts, this IgG4 reaction is also found in PSC, hepatolithiasis, and cholangiocarcinoma. In particular, the differentiation between IgG4-SC and cholangiocarcinoma is an important clinical issue. Moreover, carcinogenesis in IgG4-related diseases has been noted [1] and a few cholangiocarcinoma cases arising from IgG4-SC have also been reported [2, 3]. In this review, we focus on the IgG4 reaction in cholangiocarcinoma and the pathological IgG4-SC-induced carcinogenic features of cholangiocarcinoma.

## 2. IgG4-Related Diseases and Clinicopathological Issues

IgG4 is a minor immunoglobulin subtype that does not activate complement and comprises only 3–6% of all circulating IgG in adults [4]. Elevated serum IgG4 levels and abundant IgG4-positive plasma cell infiltration in affected organs mark IgG4-related diseases [4–6]. The physiological and pathological significance of IgG4 remains unknown in both healthy individuals and IgG4-related disease patients. However, IgG4 responses occur at various levels in diseases not related to IgG4 including PSC [7, 8]. Moreover, IgG4-SC and type I autoimmune pancreatitis are characterized by sclerosing lesions (storiform fibrosis). Setting an upper normal limit for serum IgG4 to be 135 mg/dL, Hamano et al. [4] reported diagnostic sensitivity of 95% and specificity of 97% (versus pancreatic cancer) for autoimmune pancreatitis. Raina et al. [9] reported that as many as 7% of pancreatic cancer patients have serum IgG4 levels >135 mg/dL and concluded that, for patients with pancreatic mass lesions and suspected cancer, an IgG4 level between 135 and 200 mg/dL should be cautiously interpreted and rejected as the diagnostic criterion for autoimmune pancreatitis without further evaluation. Therefore, a pathological examination is necessary to differentiate IgG4-related diseases from tumors in other organs. IgG4 reactions characterized by an increase in the number of IgG4-positive cells are speculated to be nonspecific during pathological conditions including cancer and the presence of IgG4-positive cells is not a histological hallmark of IgG4-related diseases. An IgG4 reaction may simply be the result of an immunoreaction within a certain cytokine milieu and may have limited pathological significance in affected organs. Moreover, storiform-type sclerosing fibrosis is a characteristic feature of IgG4-related diseases including IgG4-SC, but cholangiocarcinomas and pancreatic cancer usually accompany some degree of desmoplastic change.

## 3. IgG4 Reaction and Its Distribution in Biliary Tract Cancers

**Figure 1:**
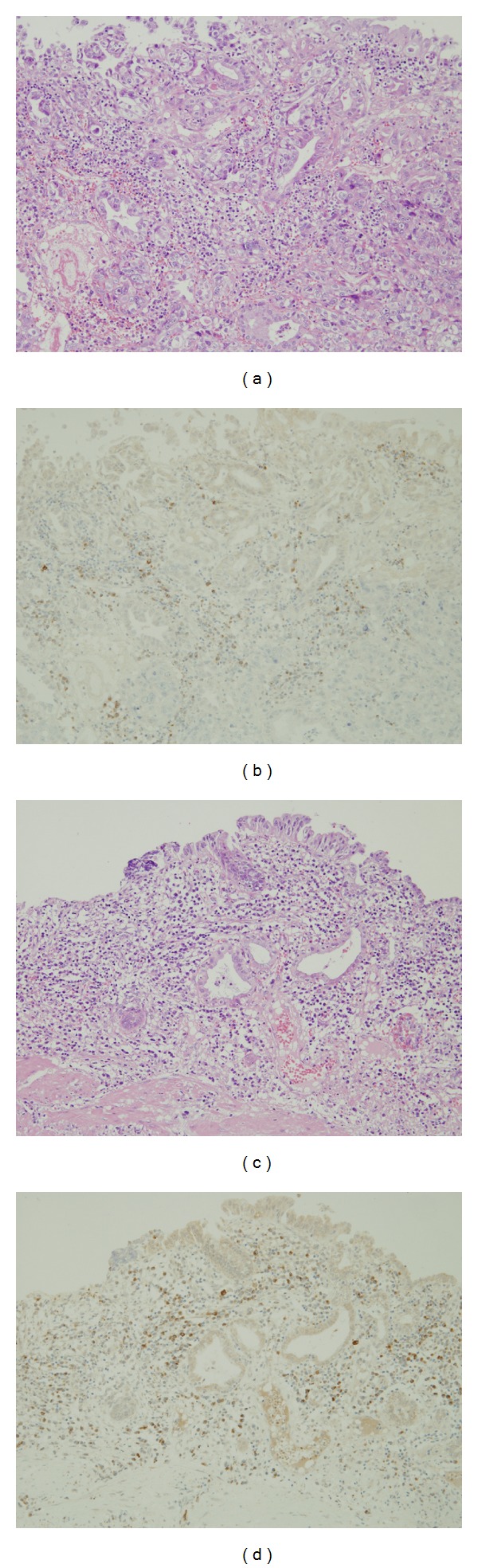
Carcinoma ((a) and (b)) and nonneoplastic ((c) and (d)) areas at the mucosal surface of gall bladder cancer. Severe inflammation ((a) and (c)) and many IgG4-positive cells ((b) and (d)) were found in both neoplastic and nonneoplastic areas. (a) and (c) hematoxylin and eosin stain (HE); (b) and (d) IgG4 immunohistochemistry.

Biliary tract cancers can be anatomically divided into intrahepatic and extrahepatic cholangiocarcinomas; the latter includes hepatic hilar cancer, common bile duct cancer, gall bladder cancer, and cancer of the papilla of Vater. The biological behavior and carcinogenicity of each cancer differ, but the histology of most biliary tract cancers is the same as that of ordinary adenocarcinomas. In addition to neoplastic lesions, several types of cholangitis causing biliary stenosis are important in the differential diagnosis of biliary diseases. PSC and IgG4-SC clinicopathologically mimic extrahepatic cholangiocarcinomas. In particular, the clinicopathological differentiation of IgG4-related diseases from neoplasms is important because desmoplastic change and, rarely, mass formation as well as marked IgG4-positive cell infiltration are found. Moreover, the IgG4 reaction often occurs in malignant neoplasms including pancreatic cancer [9–12] and the relation between cancer-related immunity and IgG4 reaction has been speculated. We surveyed the IgG4 reaction in biliary tract cancers and demonstrated that the IgG4 reaction occurs at various degrees in most cholangiocarcinomas located in the hepatic hilus, common bile ducts, papilla of Vater, and gall bladder cancers (Figures 1(a) and 1(b)) but very rarely in cholangiocarcinoma of the liver (intrahepatic cholangiocarcinoma, ICC). In addition, IgG4-SC mainly affects bile ducts in the hepatic hilus, common bile ducts, and gall bladder [13–15], indicating anatomical similarities between organs affected by IgG4-SC and biliary tract cancers with the IgG4 reaction. In biliary tract cancers, except ICC, 43% cases exhibited >10 IgG4-positive plasma cells/hpf, clinical diagnostic criteria of IgG4-SC 2012 [16], and 9% cases exhibited marked infiltration of IgG4-positive cells over 50/hpf. Moreover, Resheq et al. [17] recently showed that six out of 19 (32%) patients with hilar cholangiocarcinoma were IgG4-positive (≥20 IgG4-positive plasma cells/hpf). However, the authors concluded that IgG4-positive plasma cells in combination with clinical parameters as criteria to distinguish hilar cholangiocarcinoma from IgG4-SC had limited utility and may be misleading under conditions when malignancy is not diagnosed.

The IgG4 reaction is scattered within and around cancerous nests. IgG4-positive cells are particularly prominent around nests, invasive areas facing noncancerous biliary walls, and fibroadipose tissue and are interspersed with other inflammatory cells. Moreover, in biliary tract cancer with a marked IgG4 reaction, the surrounding nonneoplastic biliary mucosa and the carcinoma area are often accompanied with an IgG4 reaction (Figures 1(c) and 1(d)). One characteristic feature of IgG4-related diseases, the perineural infiltration of IgG4-positive cells (also a characteristic feature of IgG4-SC), is mostly found in cases with the IgG4 reaction, suggesting neurotropic IgG4-positive cells irrespective of IgG4-SC and biliary tract cancer with the IgG4 reaction. However the significance and mechanism of perineural infiltration of IgG4-positive cells are unknown. Obliterative phlebitis caused by IgG4-positive cells and storiform-type fibrosis are characteristic features of IgG4-SC. However, these findings have to be differentiated from vascular invasion by cancer cells and desmoplastic change in extrahepatic cholangiocarcinomas.

In the advanced cholangiocarcinoma cases, the presence of the cholangiocarcinoma arising from IgG4-SC is speculated, but it is pathologically hard to differentiate between cholangiocarcinoma with IgG4 reaction and cholangiocarcinoma arising from IgG4-SC. However, the presence of IgG4-SC cases accompanying biliary precancerous lesion indicates the possibility of biliary carcinogenesis arising from IgG4-SC.

## 4. Pathological Significance of the IgG4 Reaction in Biliary Tract Cancer

**Figure 2:**
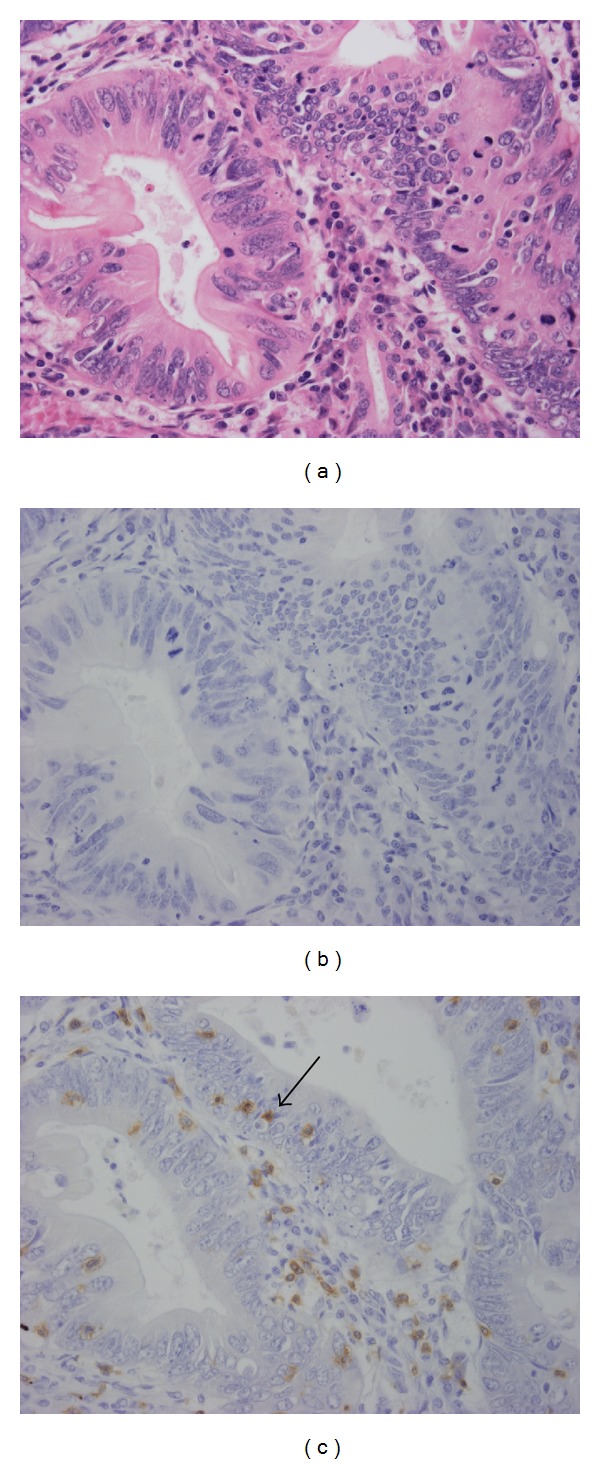
Extrahepatic cholangiocarcinoma with scant IgG4 reactions. Hematoxylin and eosin stain of inflammatory cells in the stroma of adenocarcinoma (a). Immunohistochemistry identified no IgG4-positive cells (b), but CD8-positive T cells were scattered throughout the stroma and cancerous nests (arrows, (c)).

Some pancreatic cancer cases accompanied by the IgG4 reaction and/or elevated serum IgG4 levels [9–12] in addition to cases with pancreatic cancer or cholangiocarcinoma result from IgG4-related autoimmune pancreatitis or IgG4-SC, respectively [3, 18], suggesting an association between cancer-related immunity and the IgG4 reaction. During the carcinogenesis of pancreatic cancer, the number of Foxp3-positive regulatory T cells (Treg cells) increases, whereas that of cytotoxic CD8-positive cells decreases, suggesting that Treg cells are involved in immune response control against pancreatic cancers that evade tumor-associated immune surveillance [19]. Treg cells inhibit anticancer immunity via the production of regulatory cytokines, such as IL-10 and TGF-*β*. High Treg cell frequency is speculated to reflect a poor prognosis in pancreatic and colon cancer patients [19, 20]. In addition, Treg cells play an important role in the histogenesis of IgG4 reaction in IgG4-related diseases [21–25], suggesting that these cells perform a similar function in the pathogenesis of IgG4-SC and carcinoma. We studied the association of the IgG4 reaction versus Treg and CD8-positive T cells in biliary tract cancers and demonstrated that numerous Treg cells accompanied cases with a marked IgG4 reaction. CD8-positive cytotoxic T cells (CTLs) were scattered to various degrees, irrespective of their location within or around cancer nests. CTLs mark immune activity against cancers and invade cancerous nests resembling intraepithelial lymphocytes (IELs) that are found in nonneoplastic biliary epithelial layers of biliary diseases such as primary biliary cirrhosis [26]. Consequently, patients with many CD8-positive CTLs resembling IELs showed scant IgG4 reactions (IgG4-poor cases, Figure 2). In contrast, IgG4-rich cases have few CD8-positive CTLs and a poor prognosis compared with IgG4-poor cases [27]. In other words, it is suggested that the IgG4 reaction showed positive and negative correlation with Treg cells and CTLs, respectively, signifying that immune surveillance evasion was associated with CTLs through Treg cell regulatory function.

## 5. Mechanisms of the IgG4 Reaction in Biliary Tract Cancers

**Figure 3:**
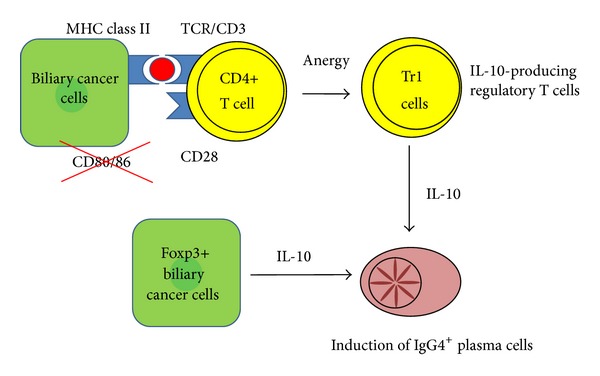
Proposed mechanisms for the induction of IgG4-positive plasma cells in cholangiocarcinoma.

Th2-type cytokines, IL-4 and IL-10, are important in the pathogenesis of IgG4-related diseases including IgG4-SC. Treg cells are characterized by the production of IL-10 and TGF-*β* and are involved in the IgG4 reaction [22, 25, 28]. In particular, IL-10 (a regulatory cytokine mainly produced by Treg cells), Th2 cells, and IL-10-producing regulatory T cells could be thought to induce the differentiation of IgG4-positive plasma cells or promote B cell switching to IgG4 in the presence of IL-4 [28, 29]. Several carcinoma tissues and cultured cancer cell lines demonstrate the expression of Foxp3 and IL-10, suggesting that cancer cells induce the Treg cell-like immunoregulatory milieu to evade immunosurveillance [30–33]. We describe two mechanisms where cholangiocarcinoma cells directly participate in the histogenesis of IgG4 reactions via cytokine milieu of IL-10 (Figure 3).

## 6. Cholangiocarcinoma Cells as Nonprofessional Antigen Presenting Cells (APCs)

**Figure 4:**
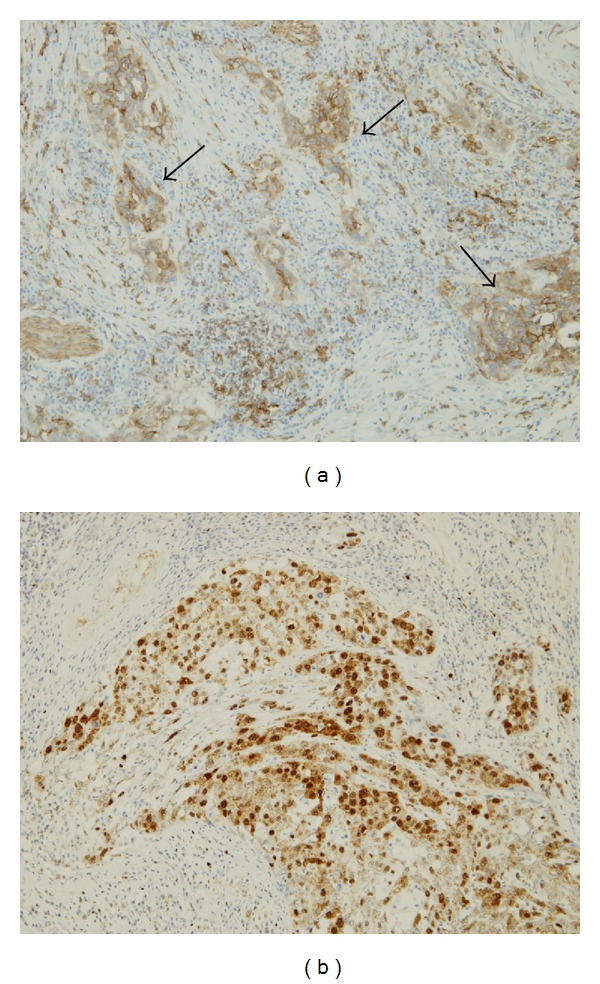
Immunohistochemistry for MHC class II (HLA-DR) (a) and the N-terminus of Foxp3 (b) in biliary tract cancers. In addition to infiltrating mononuclear cells, carcinoma cells are positive for HLA-DR ((a), arrows). The antibody reacting with the N-terminus of Foxp3 (b) highlights the nucleus and cytoplasm of cholangiocarcinoma cells.

Professional APCs such as dendritic cells expressing both MHC class II and the costimulatory molecules CD80 (B7-1) and CD86 (B7-2) present antigens with costimulatory molecules to CD4-positive T cells and differentiate from CD4-positive cells into Th1, Th2, TH17, or Treg cells depending on cytokine milieu. However, MHC class II-positive cells lacking costimulatory molecules induce anergy in native T cells. T regulatory type 1 cells (Tr1 cells) are characterized by the production of IL-10 and are induced by immature dendritic cells [34]. Moreover, costimulation-dependent T cell clones stimulated without the costimulatory signal do not proliferate but instead differentiate into IL-10-producing anergic T cells in primary biliary cirrhosis [35]. Immunocompetent cells like dendritic cells and nonimmunocompetent cells including carcinoma and normal epithelial cells express MHC class II and may present antigens. However, MHC class II-positive epithelial cells (also referred to as nonprofessional APCs) differ from professional APCs such as dendritic cells. MHC class II-positive cells that do not express the costimulatory molecules CD80 (B7-1) and CD86 (B7-2) induce IL-10-producing anergic T cells or Tr1 cells from native T cells [34, 35]. Several studies suggest that antigen presentation by MHC class II-positive epithelial cells that lack costimulation signals, for example, keratinocytes and pancreatic islet cells, promotes anergic T cells generation [36–38]. In biliary tract cancers, carcinoma cases expressing MHC class II but lacking costimulatory molecules (CD80 and CD86) are found in 54% (Figure 4(a)). These biliary tract cancer cells could act as nonprofessional APCs by generating IL-10-producing regulatory T cells (anergy T cells). Furthermore, an IL-10-predominant cytokine milieu could cause the induction of IgG4-positive cells [28, 29]. In these phenotypic cases, the number of IgG4-positive cells infiltrating carcinoma tissues was higher than that in MHC class II-negative cases.

## 7. Cholangiocarcinoma Cells as Regulatory Cells

Treg cells, Th2 cells, and IL-10-producing regulatory T cells mainly produce IL-10. Although Foxp3 is a master transcription factor for Treg cells, Foxp3 and IL-10 are expressed in several carcinoma tissues and cultured cancer cell lines, suggesting that cancer cells induce the Treg cell-like immunoregulatory milieu to evade immunosurveillance [30–33]. To validate the hypothesis that cholangiocarcinoma cells themselves function in immunosuppression similar to Treg cells, we examined the expression of Foxp3 in biliary tract cancer. The antibody reacting with the N-terminus of Foxp3 highlighted carcinoma cells and Treg cells in 39% of biliary tract cancers while the antibody reacting with the C-terminus of Foxp3 detects only the mononuclear cells that correspond to Treg cells (Figure 4(b)). Moreover, the number of IgG4-positive cells is significantly higher in Foxp3-positive than Foxp3-negative cases. This discrepancy between antibodies against different antigenic sites of Foxp3 suggests the presence of Foxp3 splice variants in cholangiocarcinoma cells. RT-PCR demonstrated that the cholangiocarcinoma cell line HuCCT1 expresses Foxp3 mRNA. Further examination of cholangiocarcinoma cells with four primer sets revealed a Foxp3 splice variant lacking exon 3 that caused a frameshift at the C-terminus creating a novel amino acid, which has been reported in a melanoma cell line [32]. Furthermore, RT-PCR and ELISA revealed that HuCCT1 cells express IL-10 mRNA and secrete IL-10 protein into the culture medium. Foxp3 expression is closely correlated with the expression of IL-10 in all Foxp3-positive cell lines [33]; however, its function as a transcription factor requires further investigation. In conclusion, cholangiocarcinoma cells perform immunosuppressive functions similar to Treg cells via IL-10 production and possibly induce the differentiation of IgG4-positive plasma cells in biliary tract cancers.

As mentioned above, cholangiocarcinoma cells are nonprofessional APCs and/or regulatory cells that directly induce IgG4 reactions in an IL-10-predominant cytokine milieu. Although the IgG4 reaction in biliary tract cancers and IgG4-SC are closely associated with the IL-10 regulatory cytokine milieu, it is possible that both mechanisms are specific for cancer tissues but different from IgG4-related pathogenesis.

## 8. Carcinogenesis in Patients with IgG4-SC

Patients with autoimmune pancreatitis occasionally have other types of cancer including pancreatic cancer [39–41] and bile duct cancer [3]. These patients are at an increased risk for various cancers and it is suggested that autoimmune pancreatitis may develop as a paraneoplastic syndrome in some patients [1, 42]. Pancreatic and biliary cancers have been reported in IgG4-related diseases [3, 11, 18], although the cause-and-effect relationship between IgG4 reactions and cancer is unknown. A study by Shiokawa et al. [1] reported that 18 cancers in various organs were found in 15 out of 108 autoimmune pancreatitis patients (13.9%), during a median follow-up period of 3.3 years. The relative risk of cancer was 4.9 for the autoimmune pancreatitis patients on diagnosis. Before autoimmune pancreatitis patients initiated corticosteroid therapy, numerous IgG4-positive plasma cells were observed in the cancer stroma; no patient had an autoimmune pancreatitis relapse after successful cancer treatment. Therefore, it was concluded that autoimmune pancreatitis may have developed as a paraneoplastic syndrome in some patients. In contrast, Hirano et al. [43] surveyed 113 patients with IgG4-related disease in whom malignancy was not diagnosed at the time of IgG4-RD onset and revealed that the incidence of the observed malignancies was not significant, compared with the expected incidence in an age- and sex-matched general Japanese population.

PSC and IgG4-SC target large bile ducts such as the hepatic hilar bile duct and extrahepatic bile ducts. Both diseases exhibit similar clinicopathological behaviors, that is, bile duct stenosis and biliary obstruction. PSC often develops into cholangiocarcinoma (incidence 7–15%, annual incidence of 0.5–1.5%) [44, 45]. The relationship between cholangiocarcinoma and IgG4-SC is unclear, although a few cases of IgG4-SC are associated with cholangiocarcinoma or its precursor lesion [2, 46]. The World Health Organization (WHO) defines biliary intraepithelial neoplasia (BilIN) as a cholangiocarcinoma precursor that is classified according to morphological atypia into the three subtypes BilIN1, BilIN2, and BilIN3 [47]. BilIN3* in situ* carcinoma directly contributes to the progression of overt invasive cholangiocarcinomas. BilIN3 as well as BilIN1-2 is found in IgG4-SC as well as PSC, suggesting a risk for invasive cholangiocarcinoma progression in IgG4-SC. In the IgG4-SC case, moreover, the BilIN lesion expressed a mutated form of the p53 tumor suppressor protein [46], suggesting that cholangiocarcinoma is possibly associated with IgG4-SC as precursors of malignancy. Therefore, note the possibility that cholangiocarcinoma is associated with IgG4-SC during IgG4-SC diagnosis and IgG4-SC treatment.

Two mechanisms are possible regarding cooccurrence of cancer and autoimmunity: (1) sustained inflammation in the presence of an autoimmune disease is considered to create immunological environments favorable for cancer development and (2) cancers may induce autoimmune diseases as a paraneoplastic syndrome [1]. Although it is unknown whether IgG4-related diseases including IgG4-SC are true autoimmune diseases, an immune dysfunction including autoimmunity is surely associated with the pathogenesis of IgG4-related diseases. As mentioned above, the regulatory cytokine IL-10 induces the differentiation of IgG4-positive plasma cells, promotes the conversion of B cells into IgG4 in the presence of IL-4 [28, 29], and closely associates with the pathogenesis of IgG4-related diseases [25, 48]. IL-10 is a regulatory cytokine that broadly functions as an immune inhibitory cytokine to support tumor growth. This suggests that Treg cells play a role in the progression and metastasis of various malignant tumors, particularly for controlling the immune responses against carcinomas from the premalignant stage until established cancer [19, 49]. Therefore, in IgG4-SC, an IL-10-related cytokine milieu initiates the IgG4 reaction and also suppresses tumor-reactive T cells, suggesting that IgG4-SC may accelerate cholangiocarcinoma development. In conclusion, IL-10-based regulatory cytokine networks evade host immune responses in cancer patients with IgG4-related diseases and further suggest an association with cholangiocarcinoma.

## 9. Differential Diagnosis 

IgG4-SC complicated autoimmune pancreatitis can be differentiated by the same diagnostic criteria as autoimmune pancreatitis, such as serum IgG4 levels and lesion distribution. However, differentiating IgG4-SC cases without pancreatic and other organs involvement from conditions such as PSC and cholangiocarcinoma is challenging. Moreover, the mean IgG4 serum level is relatively lower in IgG4-SC cases without autoimmune pancreatitis than in cases with autoimmune pancreatitis. Furthermore, hilar hepatic lesions that resemble hepatic hilar cholangiocarcinoma frequently accompany IgG4-SC cases without autoimmune pancreatitis [50, 51]. Bile duct biopsy and cytological examination are particularly important to exclude malignancies. At present, >40% IgG-positive plasma cells and >10 cells/hpf of biopsy samples are comprehensive histological diagnostic criteria for IgG4-related diseases (2011 and 2012) [16, 52–54]. However, these criteria should be applied only if malignant neoplasms are denied. Cholangiocarcinoma cases in which an inflammatory reaction is characterized by large numbers of IgG4-positive plasma cells within or around the tumors exist in addition to cholangiocarcinoma cases and BilIN lesions that are preceded by IgG4-SC. Moreover, other histological features of IgG4-SC, storiform fibrosis and obliterative phlebitis, can be differentiated from cholangiocarcinoma and PSC but are not located at the superficial biliary mucosa. Therefore, it is difficult to identify histological findings from small biopsies of the superficial bile duct mucosa and impossible to completely exclude cholangiocarcinoma during IgG4-SC diagnosis by biopsy and cytology. Multiple biopsies and specimens from the same site may be needed to identify cancerous or atypical cells [4]. Moreover, biopsies from the papilla of Vater [55] and liver [56] are useful for IgG4-SC diagnosis.

## 10. Concluding Remarks

In this review, we described the IgG4 reaction in cholangiocarcinoma. Correlations between IgG4 and malignant neoplasms are noted; however, their cause-and-effect relationship needs further clarification. Furthermore, a prospective study is needed to elucidate the role of IgG4-related diseases in carcinogenesis. At this moment, the most important procedure is ruling out cancer in the diagnosis of IgG4-SC. We, pathologists, have to know the IgG4 reaction in cholangiocarcinoma from the aspect of the presence of IgG4-positive cells, its density, and its distribution in order to avoid the rapid diagnosis that the presence of IgG4-positive cells in biliary mucosa is of help as evidence of IgG4-SC.
